# Supplementary material for: Robust detection of translocations in lymphoma FFPE samples using targeted locus capture-based sequencing
Source: Nat Commun. 2021 Jun 7;12:3361. doi: 10.1038/s41467-021-23695-8 (PMC8184748; doi:10.1038/s41467-021-23695-8)
Supplement: Supplementary file 1 — Description of Additional Supplementary Files [file 41467_2021_23695_MOESM1_ESM.pdf]

Title: Supplementary Data 1:
Description: Probe details used in this study.

Title: Supplementary Data 2:
Description: Overview of rearrangements identified by PLIER. Rearrangements that are additionally confirmed by identification of fusion-reads are bold faced and also marked by a star “*”. Cells with “red” background indicate disagreement with FISH results. Cells with pink background indicate the calls that were denoted as “irrelevant” using butterfly plots. Cells with a yellow background refer to calls for which no FISH result was available. Calls with a blue font indicate calls for which no FISH experiment is done.

Title: Supplementary Data 3.
Description: Fusion-reads detected in this study.

Title: Supplementary Data 4:
Description: Capture-NGS translocation calls.
