# Supplementary material for: Robust detection of translocations in lymphoma FFPE samples using targeted locus capture-based sequencing
Source: Nat Commun. 2021 Jun 7;12:3361. doi: 10.1038/s41467-021-23695-8 (PMC8184748; doi:10.1038/s41467-021-23695-8)
Supplement: Supplementary file 2 — Supplementary Information [file 41467_2021_23695_MOESM2_ESM.pdf]

Robust detection of translocations in lymphoma FFPE samples using Targeted Locus Capture-based sequencing

Supplementary Methods

Step-by-step FFPE-TLC protocol

Deparaffinization buffer:
1x CutSmart buffer (Invitrogen) containing 0.02% Igepal

10x Ligation buffer:
660 mM Tris pH 7.5, 50 mM MgCl2, 50 mM DTT, 10 mM ATP

Starting material
Starting material of the FFPE-TLC procedure are single 2-10 µm coupes of regular diagnostic FFPE material, either as a scroll in a 1.5 ml Eppendorf tube or on a diagnostic microscope slide. Material from a slide should be scraped off and transferred to a 1.5 ml Eppendorf tube before starting.

FFPE-TLC, Deparaffinization

1. Add 800 µl Deparaffinization buffer to the Eppendorf tubes containing the FFPE material.
2. Heat the samples for 3 min at 80°C while shaking at 900 RPM.
3. Centrifuge 2 min, 10.000xg at 40°C.
4. Remove the paraffin layer using a pipet tip.
5. Repeat step 2-4 once.
6. Remove ~700 µl of the supernatant.
7. Add 6 µl 10% SDS.
8. Transfer the tissue with the buffer to a 130 µl Covaris Screw Cap microTUBE.
9. Sonicate the samples on a Covaris M220 for 300 seconds, Duty factor 20%, Power 75 Watts, 200 cycles/burst at 20°C.
10. Transfer the samples back to the same 1.5 ml Eppendorf tube.
11. Add 70 µl 1x CutSmart buffer.
12. Incubate 2 hours at 80°C while shaking at 900 RPM.
13. Add 30 µl 20% Triton X-100.
14. Incubate 30 minutes at 37°C while shaking at 900 RPM.

FFPE-TLC, Digestion

15. Add 100U NlaIII (New England Biolabs) and incubate 1 hour at 37°C while shaking at 900 RPM.
16. Inactivate NlaIII for 25 minutes at 65°C.

FFPE-TLC, Ligation

17. Cool the sample to RT.
18. To the 240 µl digested material, add 40 µl 10x Ligation buffer, 5 µl T4 DNA ligase and 115 µl deionized water.
19. Incubate 2 hours at RT while tumbling.

20. Add 40 µl 5M NaCl, 8 µl 10% SDS and 10 µl Proteinase K (Roche) 10 mg/ml.
21. Incubate 1 hour at 56°C and 16 hours at 80°C.

FFPE-TLC, Purification

22. Add 5 µl NucleoMag P-beads (Marcherey Nagel) and 453 µl Isopropanol.
23. Incubate 30 minutes at RT while tumbling.
24. Place in magnetic rack until beads are separated.
25. Remove supernatant by decanting and add 800 µl 80% Ethanol.
26. Remove tubes from magnet and resuspend the beads by shaking.
27. Repeat steps 24 and 25 once.
28. Remove remaining Ethanol using a pipet.
29. Air dry for 5 minutes.
30. Add 100 µl 10 mM Tris pH 7.5 and place at 55°C for 5 minutes while shaking at 900 RPM.
31. Place in magnetic rack until beads are separated.
32. Transfer the supernatant containing the reshuffled DNA to a fresh tube.
33. Measure the DNA concentration using Qubit (Invitrogen)

KAPA library prep, Fragmentation, end-repair and A-tailing

This library prep step uses the KAPA hyper prep kit (Roche) cat.07962363001

34. Take 100 ng reshuffled FFPE DNA and fill to 50 µl in a 50 µl Covaris Screw Cap microTUBE.
35. Fragment the DNA on a Covaris M220 for 90 seconds, Duty factor 10%, Power 75 Watts, 200 cycles/burst at 20°C
36. Transfer the sonicated sample to a 200 µl PCR tube
37. Make Master mix: (Kapa end repair & A tailing BUFFER) 7 µl + (Kapa end repair & A tailing ENZYME MIX) 3 µl.
38. To each 50 µl fragmented sample Add 10 µl of the master mix from former step.
39. Mix and incubate 30 minutes at 20°C and 30 minutes at 65°C, then hold at 8°C.
40. For adapter ligation, make a master mix of MilliQ 5 µl, KAPA ligation buffer 30 µl, KAPA DNA ligase 10 µl.
41. Add 5 µl KAPA dual indexed adapter (15 µM) to the sample from step 36.
42. Now add 45 µl ligation master mix (from step 7) to the sample from step 38.
43. Mix thoroughly and incubate at 20°C for 15 minutes.
44. Add 88 µl Ampure XP beads.
45. Incubate 5 minutes.
46. Wash 2 times, on magnet, with 200 µl 80% Ethanol.
47. Pipet as dry as possible, airdry for 5 minutes.
48. Elute in 53 µl EB and transfer 50 µl eluate to a fresh tube.

KAPA library prep, Double sided size selection.

49. Add 35 µl Ampure XP to the 50 µl.
50. Mix and incubate 5 minutes at RT to allow fragments larger than 450 bp to bind.
51. Place the samples on a magnet and incubate until the samples are clear.
52. Transfer 80 µl of the supernatant to a new tube.
53. Add 10 µl Ampure XP to the 80 µl, mix and incubate 5 minutes.
54. Wash 2 times, on magnet, with 200 µl 80% Ethanol.
55. Pipet as dry as possible
56. Air dry for 5 minutes
57. Elute in 23 µl EB and transfer 20 µl eluate to a fresh 200 µl-tube

KAPA library prep, Amplify the library.

58. Prepare a PCR master mix (per reaction) KAPA HiFi HotStart mix 25 µl and Library amplification Primer mix 5 µl.
59. Add the 30 µl PCR master mix to the 20 µl eluate from step 57.
60. Run on a thermocycler: 1x (98°C 45 sec) **11x** (98°C 15 sec, 60°C 30 sec, 72°C 30 sec) 1x(72°C 1 min), hold at 4°C.

61. Add 90 µl Ampure XP beads to the 50 µl PCR mix (1.8x).
62. Incubate 5 minutes.
63. Wash 2 times, on magnet, with 200 µl 80% Ethanol.
64. Pipet as dry as possible.
65. Airdry for 5 minutes.
66. Elute in 30 µl EB.
67. Determine the DNA concentration with Qubit HS/2 µl.

Hybrid Capture, hybridization

The hybrid Capture step uses the Roche HyperCap Target Enrichment Kit cat. 8286345001 and the custom lymphoma SeqCap EZ probe pool described elsewhere in the supplementary data.

68. Mix equal amounts (by mass) 16 to max 18 of each of the amplified DNA sample libraries from step 66 to obtain a single pool with a combined mass of **2 µg**.
69. Add 5 µl COT human DNA (1 mg/ml).
70. Add 5 µl HyperCap Universal blocking oligos.
71. Determine the total volume, add 2 volumes Ampure XP and mix.
72. Incubate 10 minutes.
73. Wash 1 time, on magnet, with 190 µl 80% Ethanol.
74. Pipet as dry as possible, air dry for 5 minutes.
75. Prepare Hyb mix: 7,5 µl 2x hybridization buffer + 3 µl Hyb component A.
76. Elute, off magnet, in 10.5 µl Hyb mix, mix well, incubate 2 minutes and place back in magnet.
77. Transfer 10.5 µl to a tube containing 4.5 µl SeqCap probe pool.
78. Perform the hybridization incubation in a thermocycler using the following program with heated lid set to 10°C above block temperature: 95°C for 5 minutes, 47°C for 16 to 20 hours.

Hybrid Capture, prepare capture beads and wash buffers

79. Allow the Capture Beads to equilibrate to room temperature for 30 minutes.
80. Prepare buffers (for each wash/hyb).
81. Stringent wash buffer: 40 µl 10x stringent wash buffer + 360 µl milliQ.
82. Wash buffer I: 30 µl 10x wash buffer I + 270 µl milliQ.
83. Wash buffer II :20 µl 10x wash buffer II + 180 µl milliQ.
84. Wash buffer III :20 µl 10x wash buffer III + 180 µl milliQ.
85. Bead wash buffer: 100 µl 2.5x bead wash buffer + 150 µl milliQ.
86. Aliquot 50 µl of beads per capture into a 0.2 or 1.5 ml tube.
87. Place the tubes on a magnet. Allow the solution to clear.
88. Remove and discard the supernatant.
89. Add twice the initial volume of beads of 1X Bead Wash buffer.
90. Remove tubes from the magnetic particle collector and mix.
91. Place the tubes back on the magnet, once clear, remove and discard the liquid.
92. Repeat Steps 53 - 55 for a total of two washes.
93. After removing the buffer following the second wash, add 1X the initial volume of beads of 1X Bead Wash Buffer.
94. Remove tubes from magnetic particle collector and mix.
95. Aliquot 50 µl of resuspended beads into new tube/well for each capture.
96. Place the tubes on magnet to bind the beads. Allow the solution to clear.
97. Once clear, remove and discard the supernatant.
98. The Capture Beads are now ready to bind the captured DNA. Proceed immediately to the next step.

Hybrid Capture, Bind DNA to capture beads

99. Transfer one hybridization sample to a single prepared tube/well of Capture Beads from the previous step.
100. Mix thoroughly by vortexing for 10 seconds.

101. Bind the captured sample to the beads by placing the samples in a thermocycler set to 47°C for 15 minutes (heated lid set to 57°C).

Hybrid Capture, Heated wash (47°C)

102. Preheat 2 vials with 200 µl 1x stringent wash buffer at 47°C for use in step 79.
103. After the 15-minute incubation, remove the samples from the thermocycler.
104. Thermocycler should remain at 47°C (heated lid turned on and set to maintain 57°C) for following steps.
105. Add 100 µl of (RT) 1X Wash Buffer I to the 15 µl of Capture Beads plus bead-bound DNA.
106. Mix thoroughly by vortexing for 10 seconds.
107. Place the samples on a magnetic particle collector to capture the beads. Allow the solution to clear.
108. Once clear, remove and discard the supernatant.
109. Add 200 µl of 47°C 1X Stringent Wash Buffer to each sample.
110. Mix to homogeneity by vortexing for at least 10 seconds.
111. Place on thermocycler pre-heated to 47 °C, close lid (set to 57 °C) and incubate for 5 minutes.
112. After incubating 5 minutes, remove the sample from thermocycler and place on a magnet.
113. Allow the solution to clear.
114. Once clear, remove and discard the supernatant.
115. Repeat Steps 72-78 for a total of two washes using 1X Stringent Wash Buffer.

Hybrid Capture, RT washes

116. Add 200 µl of 1X Wash Buffer I.
117. Mix thoroughly by vortexing for 10 seconds. Ensure that the mixture is homogeneous.
118. Incubate at room temperature for 1 minute.
119. Place the samples on a magnet. Allow the solution to clear.
120. Once clear, remove and discard the supernatant.
121. Add 200 µl of 1X Wash Buffer II.
122. Mix thoroughly by vortexing for 10 seconds. Ensure that the mixture is homogeneous.
123. Incubate at room temperature for 1 minute.
124. Place the samples on a magnet. Allow the solution to clear.
125. Once clear, remove and discard the supernatant being careful not to disturb the beads.
126. Add 200 µl of 1X Wash Buffer III.
127. Mix thoroughly by vortexing for 10 seconds. Ensure that the mixture is homogeneous.
128. Incubate at room temperature for 1 minute.
129. Place the samples on a magnet. Allow the solution to clear.
130. Once clear, remove and discard the supernatant.
131. Place the samples on a magnet. Allow the solution to clear.
132. Once clear, remove and discard the supernatant.
133. Remove the samples from the magnetic particle collector.
134. Add 15 µl PCR-grade water to each tube/plate well of bead-bound DNA sample.
135. Store the beads plus captured samples at -15 to -25°C or proceed to Amplification.

Hybrid Capture, Amplification

136. Prepare a PCR master mix (per reaction) KAPA HiFi HotStart mix 25 µl and Library amplification Primer mix 5 µl.
137. Add the 30 µl PCR master mix to the 20 µl eluate from step 26
138. Run on a thermocycler: 1x (98°C 45 sec) **14x** (98°C 15 sec, 60°C 30 sec, 72°C 3 min 30 sec) 1x (72°C 1 min), hold at 4°C.
139. Add 90 µl Ampure XP beads to the 50 µl PCR mix (1.8x).
140. Incubate 5 minutes.
141. Wash 2 times, on magnet, with 200 µl 80% Ethanol.
142. Pipet as dry as possible.
143. Air dry for 5 minutes.

144. Elute in 53 µl EB, transfer 50 µl to a new tube.
145. Determine the DNA concentration with Qubit.

Capture-NGS

DNA isolation, library preparation and sequencing

DNA was extracted from 3-10 x 10 µm FFPE sections using the QIAamp DNA FFPE Tissue Kit (Qiagen, Hilden, Germany) according to manufacturer's protocol. Peripheral blood DNA was extracted using the QIAamp Blood Mini Kit (Qiagen, Hilden, Germany) according to the manufacturer's spin protocol. Isolated DNA was quantified using a Qubit 2.0 Fluorometer using the QubitBR kit (Thermo Fisher Scientific, Carlsbad CA, USA) and 250-800 ng in a total volume of 130 µl was fragmented with a Covaris S2 or ME220 (Covaris Inc, Woburn MA, USA) **(Suppl. Table 5),** for 6 minutes at 200 cycles per burst to an average size of 180-220 bp for the Covaris S2 and for 3 minutes at 1000 cycles per burst to an average size of 250-300 bp. DNA concentrations and the fragmentation profile/size distribution were determined with a 2100 bioanalyzer using the Agilent DNA 1000 kit (Agilent Technologies, Santa Clara, CA). 250 ng of 180-220 or 250-300 bp fragmented DNA was used to create NGS libraries with the KAPA library preparation kit (KAPA Biosystems, Wilmington MA, USA). In short, the DNA ends were repaired (20°C for 30 minutes) and single A-tails were ligated (30°C for 30 minutes). Subsequently, uniquely indexed adapters (Roche NimbleGen, Madison WI, USA; IDT, Coralville IA, USA) were ligated overnight (16°C) after which size selection was performed to retain fragments between 250-450 bp. DNA was amplified for seven polymerase chain reaction (PCR) cycles. An aliquot of the created DNA libraries was subjected to targeted capture. A capture panel was designed with NimbleGen design software (Roche). The capture panel covers exons of ~350 genes (~1.5 Mb) for mutation analysis and multiple chromosomal regions (including genes, introns and intergenic regions; ~1.5 Mb) for translocation analysis (Roche order ID 0200204534, ID 43712, and ID 1000002633) (**Suppl. Table 6**). Capture was performed according to NimbleGen EZ SeqCap library protocol V5.1 (Roche NimbleGen, Madison WI, USA). Per capture, DNA of eight libraries were equimolarly pooled together in one tube to a total of 1 µg DNA. Probe hybridization was performed overnight at 47°C. Pools were amplified for 14 PCR cycles. Three pools were equimolar pooled and loaded together on one sequence lane and sequenced 125 bp or 150 bp paired-end on a HiSeq 2500 or 4000 respectively (**Suppl. Table 5**) (Illumina, San Diego CA, USA).

Alignment of sequence reads

NGS reads were de-multiplexed with Bcl2fastq (Illumina). Adapters and poor quality bases were trimmed with SeqPurge (-min len 20 ; v0.1-104) . Reads were aligned against the human reference genome (hg19) with BWA mem (-M -R ; v0.7.12) [1]. Read realignment with ABRA (v0.96)[2] was used to improve alignment accuracy. The aligned BAM files were sorted on query name with Sambamba (v0.5.6), and duplicate reads were flagged with Picard tools MarkDuplicates (v2.4.1), using the setting ASSUME SORT ORDER=queryname. This setting is required to mark duplicate secondary alignments in addition to duplicate primary alignments. [3, 4]. Next, reads were sorted by coordinate (Sambamba) for compatibility with the rest of the data analysis pipeline.

Structural variant analysis

The part of the pipeline for structural variant analysis, including translocations, inversions, deletions, insertions and duplications, was created in the workflow management system Snakemake [5]. To obtain high sensitivity and specificity 4 translocation detection algorithms were combined: BreaKmer (v.0.0.4)[6], GRIDSS (v.1.4.2)[7], NovoBreak (v.1.1.3)[8] and Wham (v.1.7.0)[9]. These were selected based upon the following criteria. 1. Possibility to detect translocations 2. Functionality with paired end Illumina sequencing data of short insert size. 3. Usable on targeted sequencing data 4. Documentation

available 5. Maintained until at least 2017. BreaKmer, GRIDSS and NovoBreak were executed with default settings. Wham was executed with mapping quality of 10 (-p) and base quality of 5 (-q). For compatibility with BreaKmer, chromosome-prefixes were removed from the BAM file. BreaKmer requires a target bed file containing the regions of interest for translocation detection, to reduce assembly time and to obtain higher accuracy, the translocation targets were divided in regions of 5 kb in the target BED file.

To be able to combine the output of these tools, the output was converted in R (v.3.4.1) to be comparable between tools, and gene annotation was added. To remove noise, filters were applied. In subsequent order the following SVs were removed from the data:

1. SVs with both breakpoints off-target, further than 300 bp outside the capture probe location.
2. Duplicate SVs with exactly the same breakpoints detected with the same tool.
3. SVs not meeting set thresholds for the tool. For BreaKmer, at least 4 split reads and 3 discordant reads, for Wham at least 8 reads (sum of discordant and split reads), for GRIDSS a quality score above 450 and for NovoBreak an average coverage of at least 4 high mapping quality translocation reads.
4. SV output of the four tools were combined and SVs detected by only one tool were removed. Hence, only SVs recognized by at least 2 tools were included. Therefore, breakpoints that lie within a 10 bp margin were considered to be the same SV.

Blacklist

Examination of the results showed multiple often recurrent SVs. Visual inspection of these events in the integrative genome viewer (IGV) taught us that those SVs were artifacts of different origins. Part of the artifactual SVs were a consequence of highly repetitive regions in the genome, others were introduced by partly homologous regions. Furthermore, some common germline SVs, especially small indels, were detected in the data. To remove those problematic regions from the output, a blacklist was created based on a set of 25 non-tumor samples, (12 blood samples, 4 FFPE hyperplasia lymph node, 6 FFPE reactive lymph node and 3 FFPE epithelial tissues). For these 25 samples SV detection was performed following the exact same DNA, isolation, preparation, capture and NGS procedures as well as the four selected detection tools with the same settings. Common breakpoint locations detected in at least 2 non-tumor samples within a margin of 10 bp were added to the blacklist using Bedtools multi-inter (v0.2.17). Blacklisted areas less than 50 bp apart were merged to one region with Bedtools merge. SVs with one of the breakpoints within the blacklisted regions were removed from the SV detection output. Remaining SVs were manually inspected in IGV (**Supplementary Data 4**).

References

1. Li, H. Aligning sequence reads, clone sequences and assembly contigs with BWA-MEM. *arXiv preprint arXiv:1303.3997* (2013).
2. Mose, L.E., Wilkerson, M.D., Neil Hayes, D., Perou, C.M. & Parker, J.S. ABRA: Improved coding indel detection via assembly-based realignment. *Bioinformatics* **30**, 2813-2815 (2014).
3. Tarasov, A., Vilella, A.J., Cuppen, E., Nijman, I.J. & Prins, P. Sambamba: Fast processing of NGS alignment formats. *Bioinformatics* **31**, 2032-2034 (2015).
4. Broad Institute Picard Tools. *GitHub repository:* http://broadinstitute.github.io/picard/ (2018).
5. Köster, J. & Rahmann, S. Snakemake-a scalable bioinformatics workflow engine. *Bioinformatics* **28**, 2520-2522 (2012).
6. Abo, R.P. et al. BreaKmer: detection of structural variation in targeted massively parallel sequencing data using kmers. *Nucleic Acids Research* **43**, e19-e19 (2015).
7. Cameron, D.L. et al. GRIDSS: sensitive and specific genomic rearrangement detection using positional de Bruijn graph assembly. *Genome research* **27**, 2050-2060 (2017).

8. Chong, Z. et al. novoBreak: local assembly for breakpoint detection in cancer genomes. *Nature methods* **14**, 6-11 (2017).
9. Kronenberg, Z.N. et al. Wham: Identifying Structural Variants of Biological Consequence. *PLOS Computational Biology* **11**, e1004572-e1004572 (2015).

Structure of FFPE-TLC reads

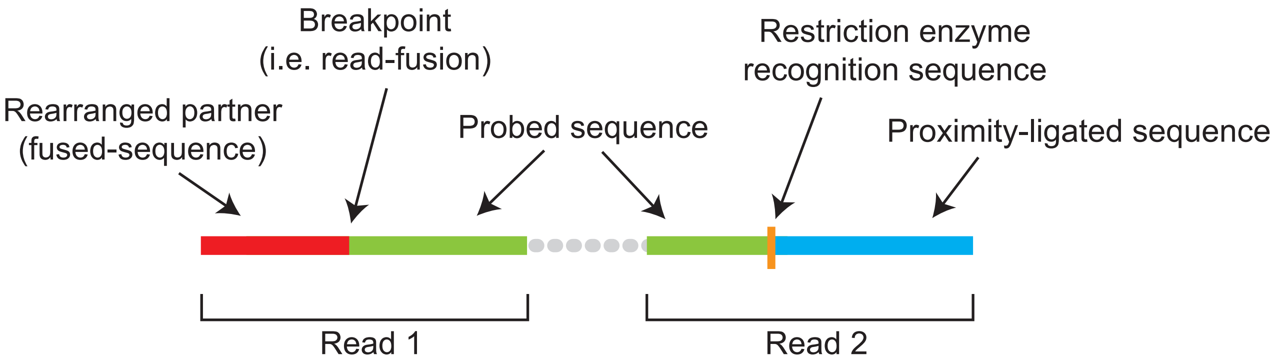

Suppl. Figure 1. Schematic view of read structure in FFPE-TLC samples

FFPE-TLC samples were Illumina sequenced in paired-end mode. Probed fragments (shown in light green) may be represented on one read-end only, or on both reads-ends. Apart from such fragments, proximity-ligation fragments (shown in blue) can be present. Such fragments are recognizable through a restriction site recognition sequence (shown as a vertical line in orange) that links them to the probed fragments. Proximity-ligation fragments may originate from the surroundings of the probed area, or from the neighborhood of the rearranged partner if a rearrangement is present either inside the probed area or in its vicinity. If a rearrangement is present, FFPE-TLC reads can also carry fragments that are produced through fusion of probed (or proximity-ligation) fragments to sequences from the rearranged partner (shown in red). Such reads can depict the rearrangement event in base pair resolution and therefore provide even further detail about the occurred structural variant.

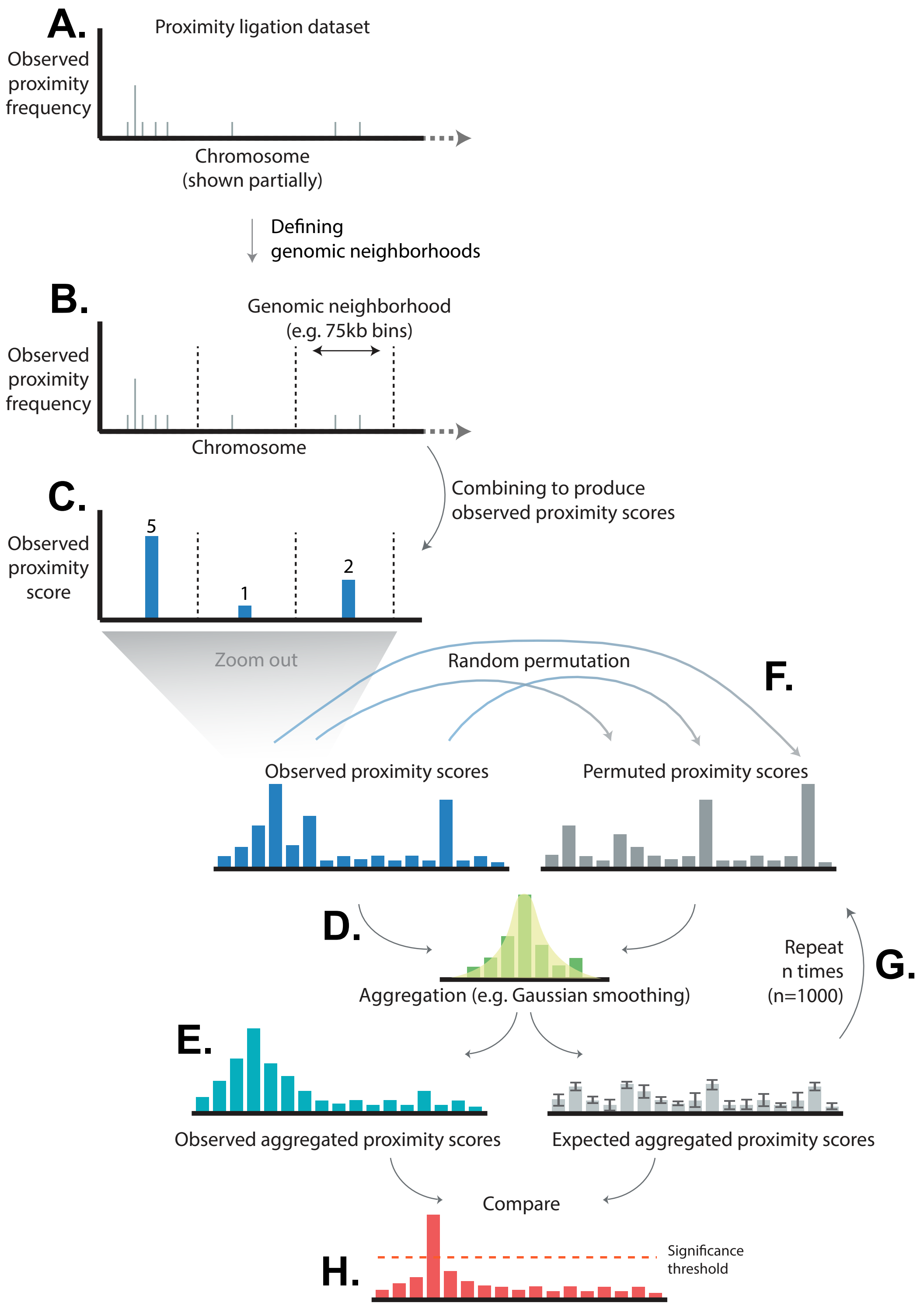

Suppl. Figure 2. Schematic view of read structure in FFPE-TLC samples

A. In a given FFPE-TLC dataset that contains mapped fragments (i.e., proximity-ligation products), B. PLIER initially splits the reference genome into equally spaced genomic intervals. C. PLIER then calculates for every interval a “proximity frequency” that is defined by the number of segments within that genomic interval that are covered by at least a fragment (or a proximity-ligation product). D. By Gaussian smoothening of proximity frequencies across each chromosome, E. observed “proximity scores” are calculated to remove very local and abrupt increase (or decrease) in proximity frequencies that are most likely spurious. F. By in silico shuffling of observed proximity frequencies across the genome followed by a Gaussian smoothing across each chromosome, G. an expected (or average) proximity score and a corresponding standard deviation (shown schematically by error bars) are estimated for genomic intervals with similar properties (e.g., genomic intervals present on trans chromosomes). H. Finally, a z-score is calculated for every genomic interval using its observed proximity score and the related expected proximity scores and standard deviation thereof. Taken together, PLIER objectively searches for genomic intervals with significantly increased concentrations of captured fragments and considers them as prime candidates for rearrangements. Please note that this figure is merely a schematic of how PLIER functions, and the depicted coverage or statistical values do not represent real data.

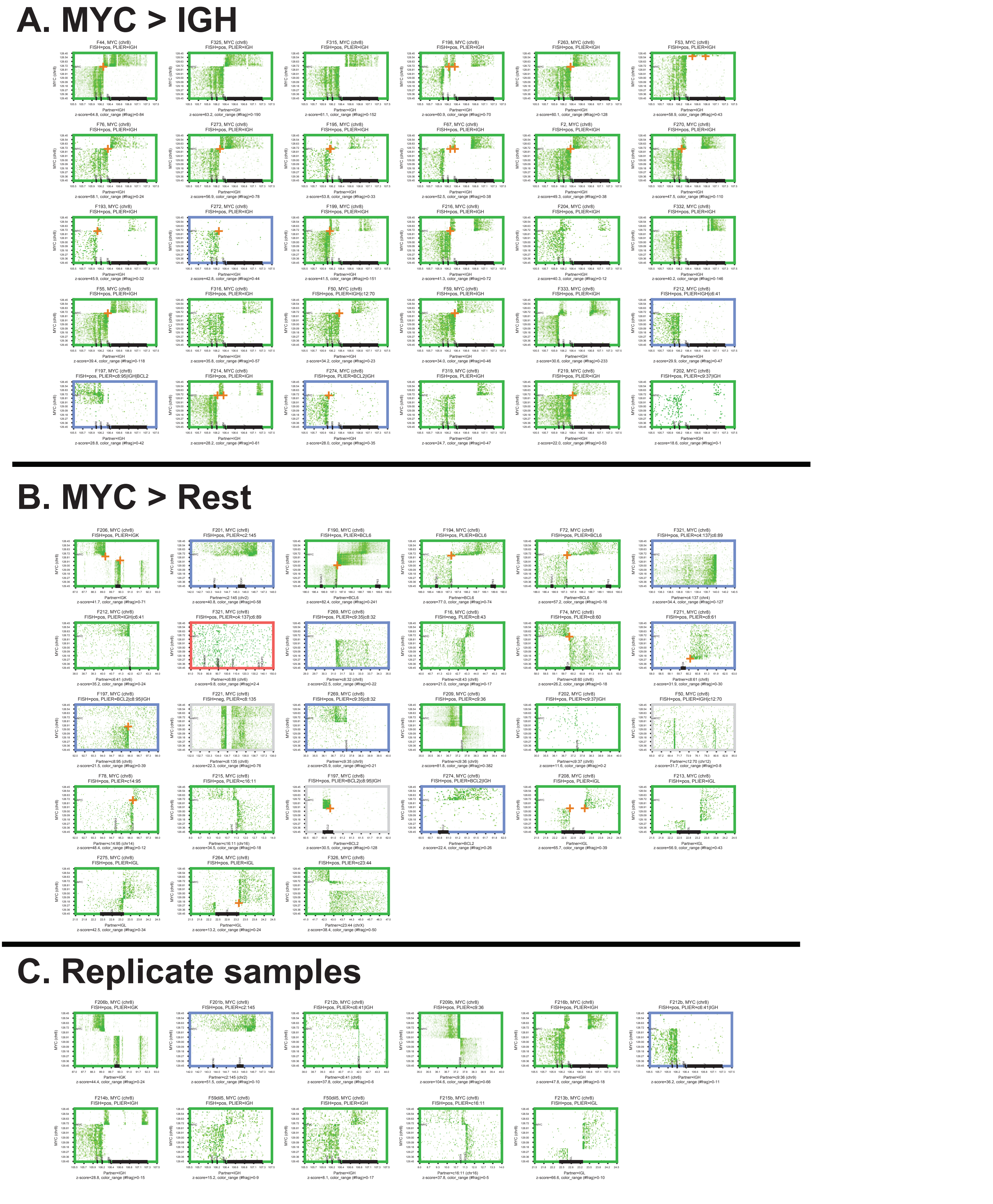

Suppl. Figure 3. Butterfly plots of rearrangements found by PLIER in MYC
Each butterfly plot is a heatmap in which the partner area is depicted on the x-axis and the probed area on the y-axis. Both axes are split into 20kb equally-spaced bins. Each element (i.e., dot) in the heatmap shows the number of reads that link the corresponding bin in the probed area to the corresponding bin in the partner area. Black boxes across axes indicate the extent of each gene. On the top of each plot patient identifier, the probed gene as well as chromosome of origin are mentioned. Underneath each plot the identified partner area, PLIER's estimated enrichment score ("z-score") and finally the minimum and maximum coverage values ("color_range") that are considered to color the heatmap from white to green are mentioned. The box around each butterfly plot indicates the type of rearrangement: reciprocal rearrangement shown in green, non-reciprocal rearrangement shown in blue, reciprocity-undetermined rearrangement shown in gray or otherwise not relevant shown in red. This classification was assigned by visually inspecting each butterfly plot to establish whether a transition of coverage (i.e., breakpoint) can be seen across the vertical axis (i.e., probed area) of the plot A. Rearrangements found from MYC to IGH across samples in our study. B. Rearrangements found between the MYC gene and any other genes across samples in our study. C. Rearrangements found in the MYC gene to any partners that are found in the replicated samples in our study.

A. BCL2 > IGH

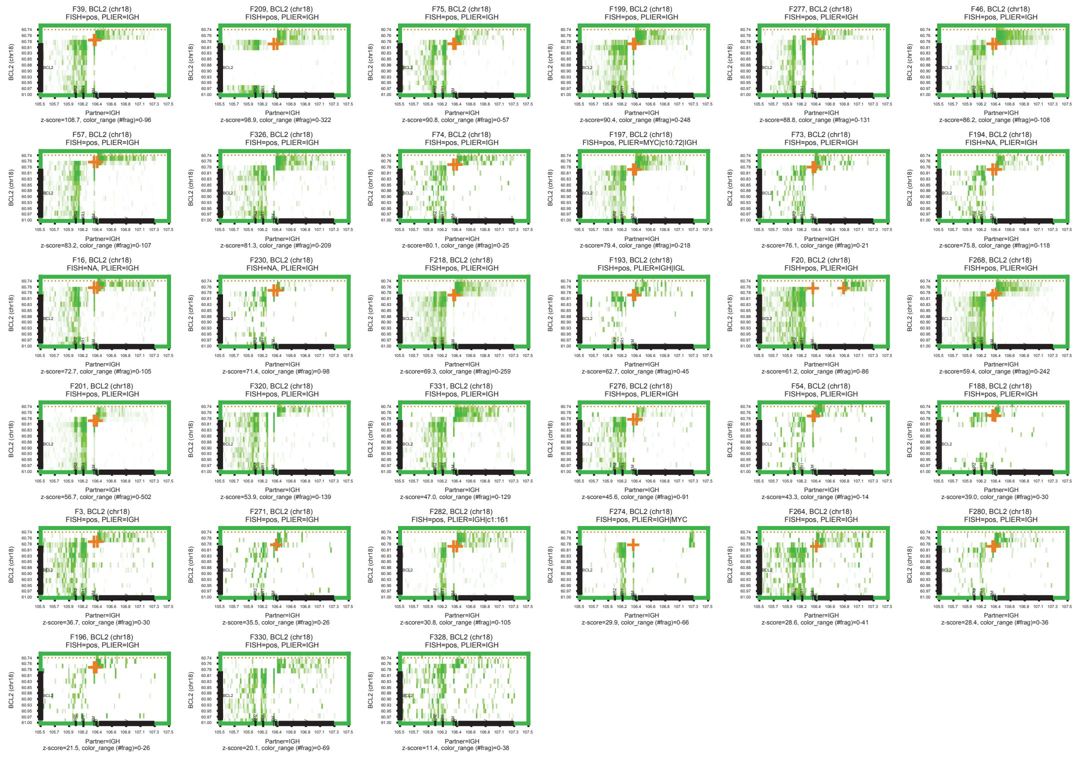

B. BCL2 > Rest

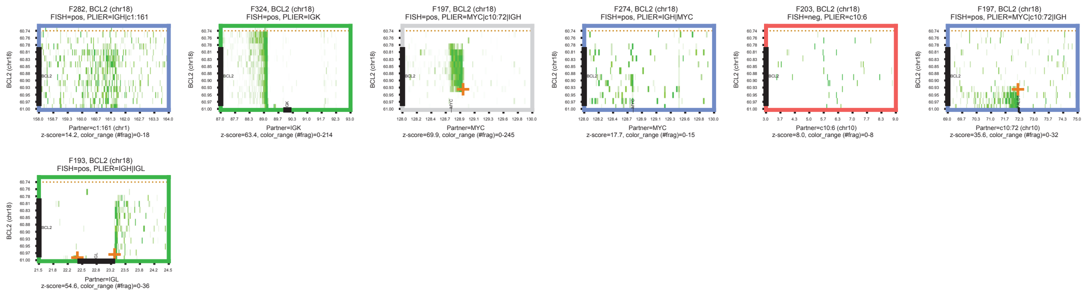

C. Replicate samples

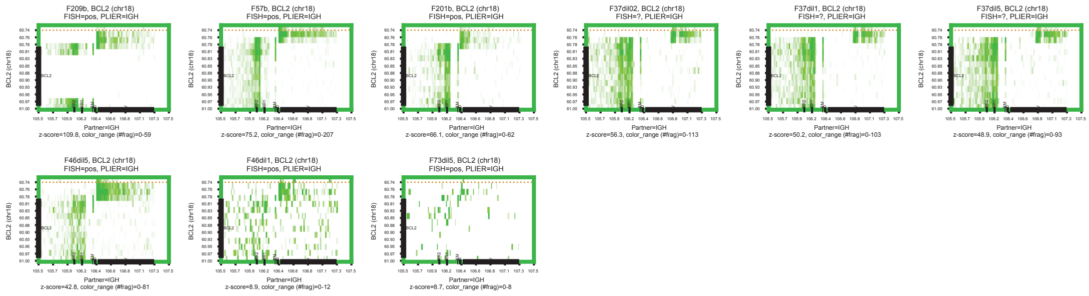

Suppl. Figure 4. Butterfly plots of rearrangements found by PLIER in BCL2
Each butterfly plot is a heatmap in which the partner area is depicted on the x-axis and the probed area on the y-axis. Both axes are split into 20kb equally-spaced bins. Each element (i.e., dot) in the heatmap shows the number of reads that link the corresponding bin in the probed area to the corresponding bin in the partner area. Black boxes across axes indicate the extent of each gene. On the top of each plot patient identifier, the probed gene as well as chromosome of origin are mentioned. Underneath each plot the identified partner area, PLIER's estimated enrichment score ("z-score") and finally the minimum and maximum coverage values ("color_range") that are considered to color the heatmap from white to green are mentioned. The box around each butterfly plot indicates the type of rearrangement: reciprocal rearrangement shown in green, non-reciprocal rearrangement shown in blue, reciprocity-undetermined rearrangement shown in gray or otherwise not relevant shown in red. This classification was assigned by visually inspecting each butterfly plot to establish whether a transition of coverage (i.e., breakpoint) can be seen across the vertical axis (i.e., probed area) of the plot A. Rearrangements found from BCL2 to IGH across samples in our study. B. Rearrangements found between the BCL2 gene and any other genes across samples in our study. C. Rearrangements found in the BCL2 gene to any partners that are found in the replicated samples in our study.

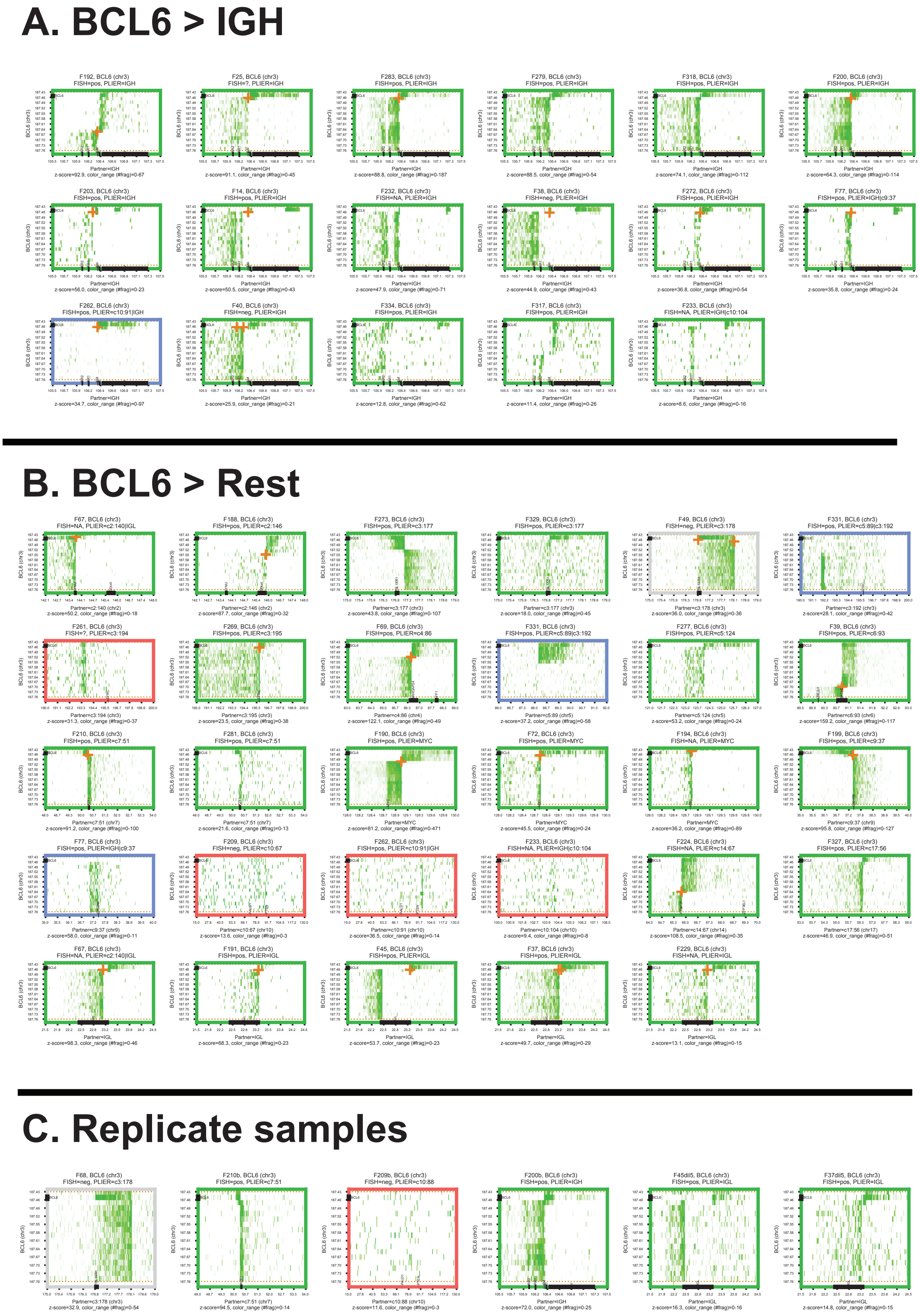

Suppl. Figure 5. Butterfly plots of rearrangements found by PLIER in BCL6
Each butterfly plot is a heatmap in which the partner area is depicted on the x-axis and the probed (target) area on the y-axis. Both axes are split into 20 kb equally-spaced bins. Each element (i.e., dot) in the heatmap shows the number of reads that link the corresponding bin in the probed area to the corresponding bin in the partner area. Black boxes across axes indicate the extent of each gene. On the top of each plot patient identifier, the probed gene as well as chromosome of origin are mentioned. Underneath each plot the identified partner area, PLIER's estimated enrichment score ("z-score") and finally the minimum and maximum coverage values ("color_range") that are considered to color the heatmap from white to green are mentioned. The box around each butterfly plot indicates the type of rearrangement: reciprocal rearrangement shown in green, non-reciprocal rearrangement shown in blue, reciprocity-undetermined rearrangement shown in gray or otherwise not relevant shown in red. This classification was assigned by visually inspecting each butterfly plots to establish whether a transition of coverage (i.e., breakpoint) can be seen across the vertical axis (i.e., probed area) of the plot A. Rearrangements found from BCL6 to IGH across samples in our study. B. Rearrangements found between BCL6 and any other genes across samples in our study. C. Rearrangements found in the BCL6 gene to any partners that are found in the replicated samples in our study.

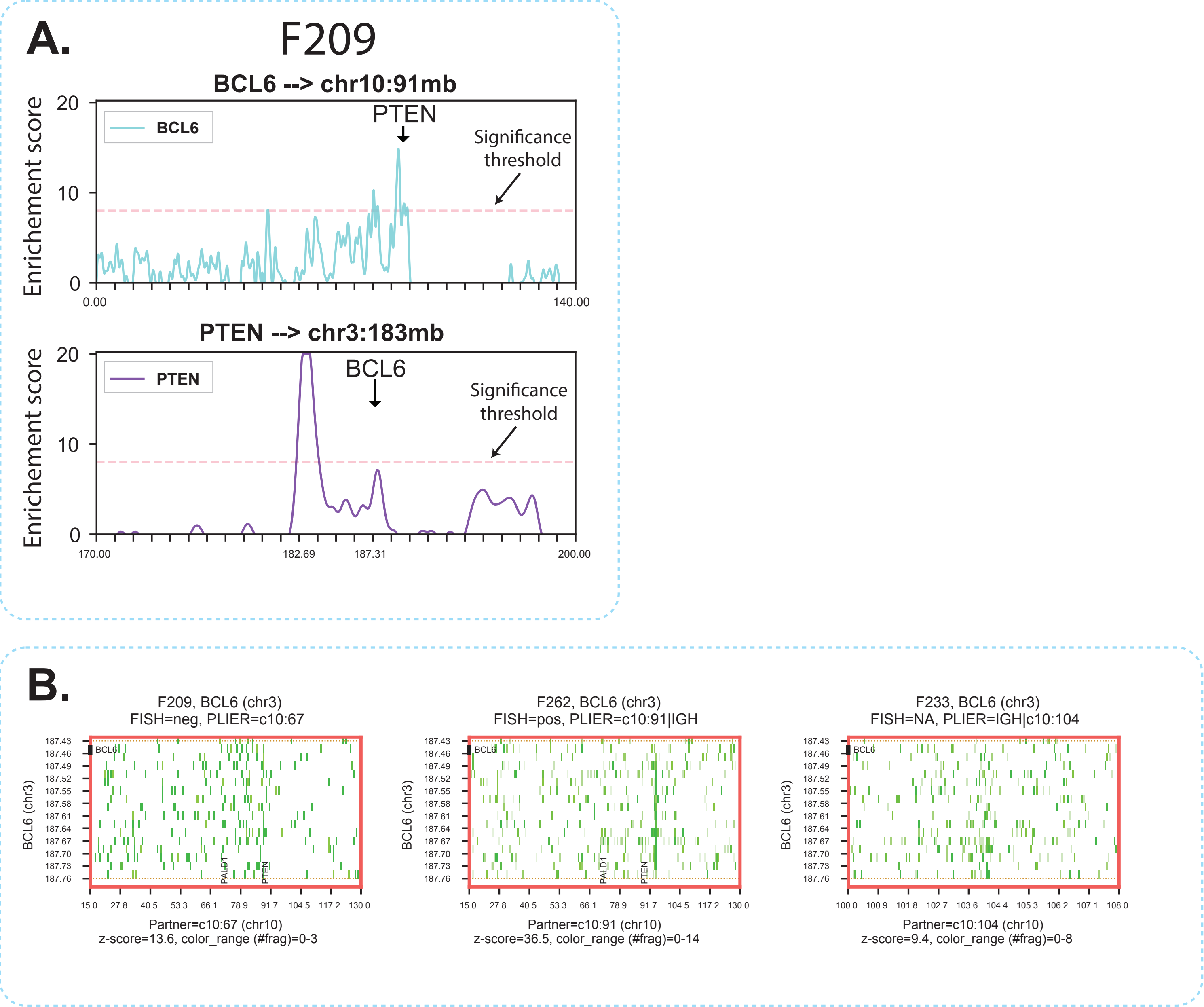

Suppl. Figure 6. Example of PLIER calls that are later identified as not relevant using butterfly plots.

A. In sample F209 when looking from BCL6, PLIER identified a significant increase of enrichment score around chr10:91mb near the PTEN gene (top plot). However, when looking from PTEN, no reciprocal peak at BCL6 was seen, but ~4.5 Mb away from BCL6. This observation confirms that the rearrangement did not occur within the region of interest (BCL6 in this case). B. The existence of not relevant cases can be further validated in a butterfly visualization of the same case (i.e., F209 looking from BCL6) that is depicted in the left most butterfly plot. As shown, no transition (or breakpoint) of coverage can be seen. Instead, a vertical pattern of coverage is visible. We observed two more cases with similar characteristics. One case was seen in F262 when looking from BCL6 and was very similar to the already described case in F209. The other case was in F233 and also looking from BCL6, but this time the increased vertical coverage was seen around chr10:104. All three cases were therefore considered as not relevant calls of PLIER.

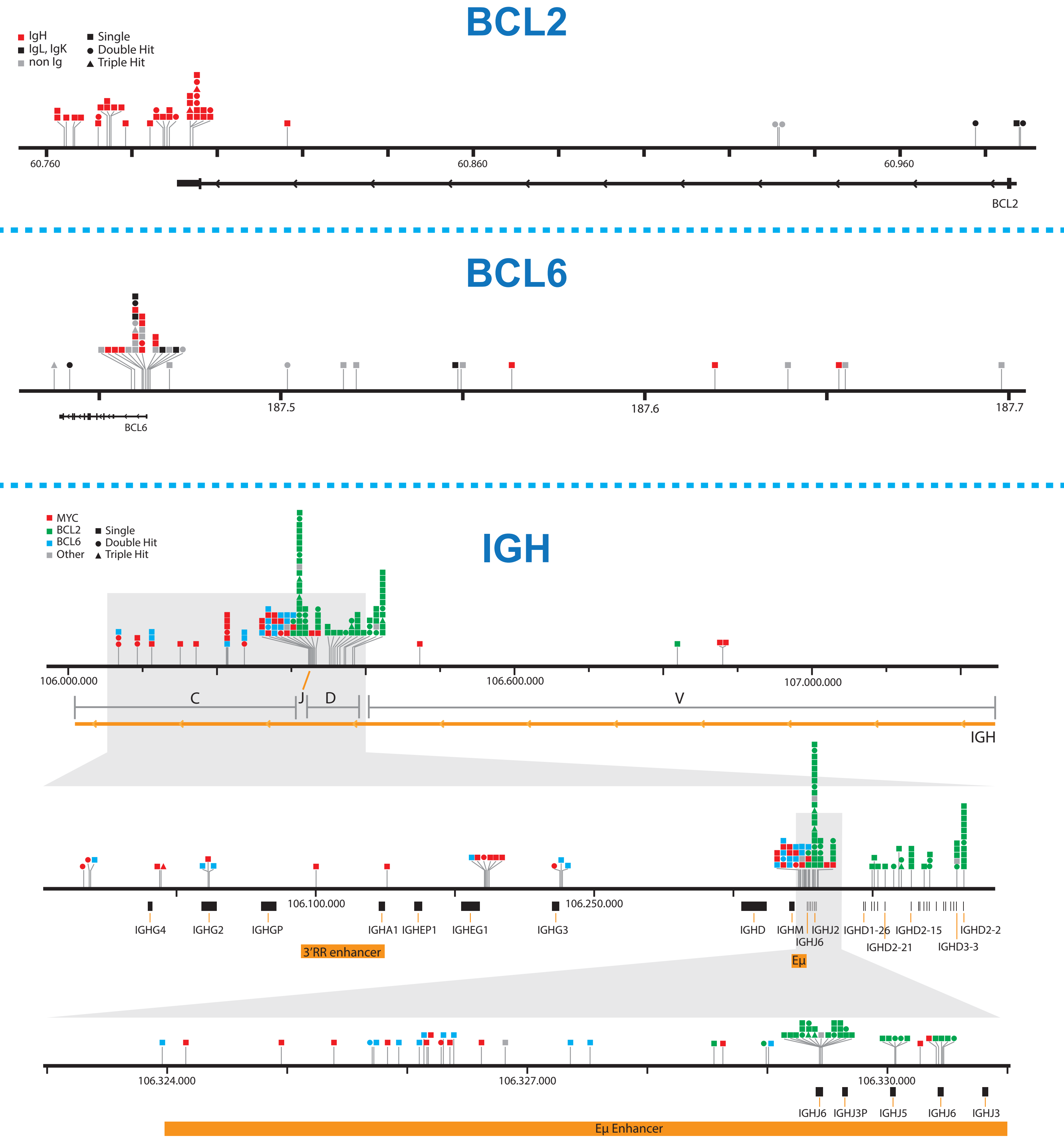

Suppl. Figure 7. Overview of breakpoints found in BCL2, BCL6 and IGH using captured fusion-reads in FFPE-TLC.
Fusion-reads in FFPE-TLC can map the occurred breakpoints of rearrangements at base pair resolution. This plot visualizes the identified breakpoints seen from BCL2, BCL6 and IGH MYC? locus, across all samples in our study.

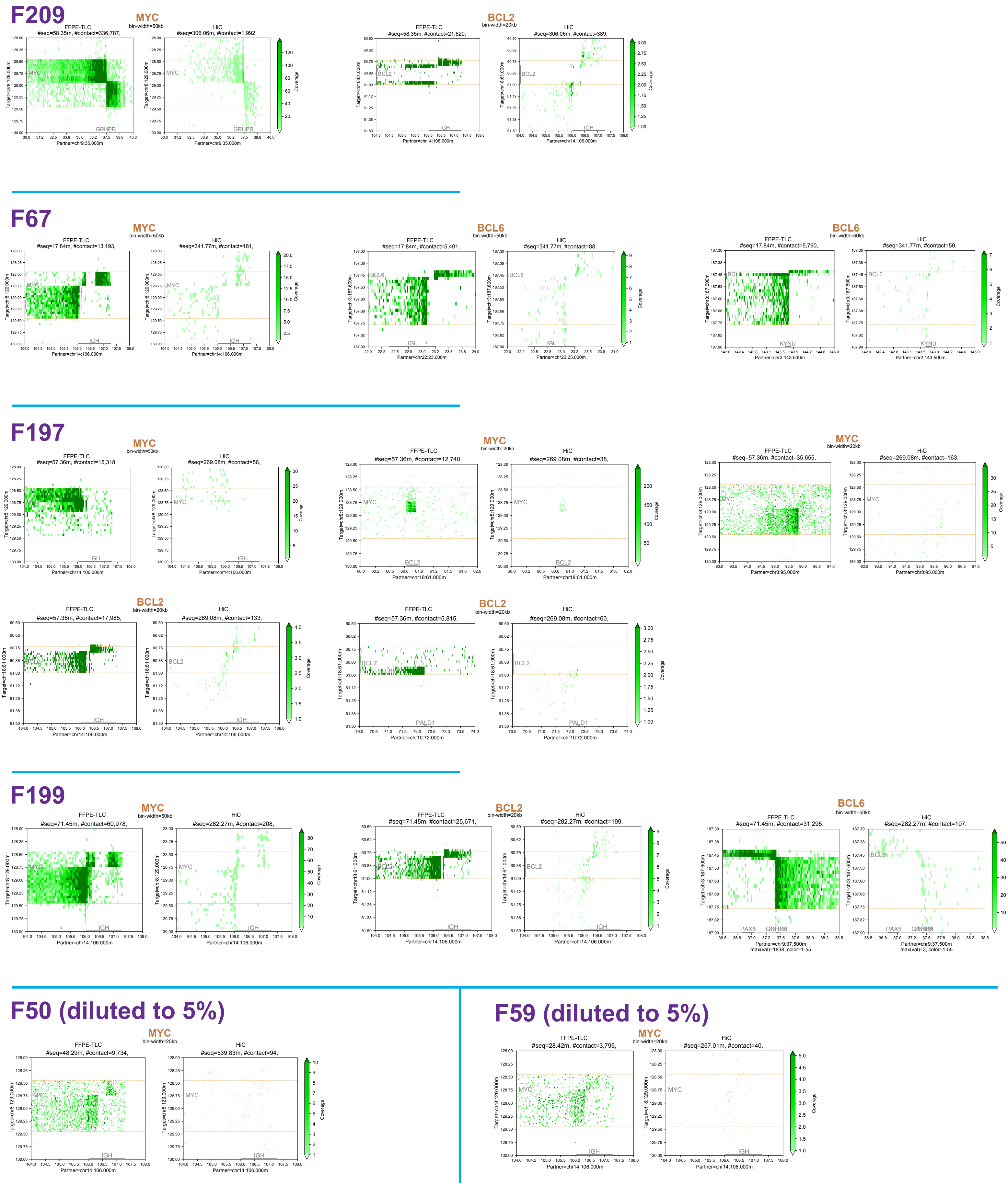

Suppl. Figure 8. Performance comparison between FFPE-TLC and Hi-C.
FFPE-TLC outperforms Hi-C in detecting every rearrangement identified across six selected samples even though the sequencing depth of the Hi-C experiment is multiple times more than the corresponding FFPE-TLC experiment. Butterfly visualization of breakpoints in six selected samples that are identified by FFPE-TLC and Hi-C. In each experiment, the material is collected from the same sample and FFPE-TLC and Hi-C are performed. In each pair of butterfly plots, the left plot is FFPE-TLC and the right plot is Hi-C. The target gene is shown in brown on the top, the bin-width used to visualize the butterfly plot is shown underneath. Each butterfly plot also includes the sequencing depth of each experiment (#seq) and the number of ligation-product that could be captured by each experiment within the visualized interval (#contact).

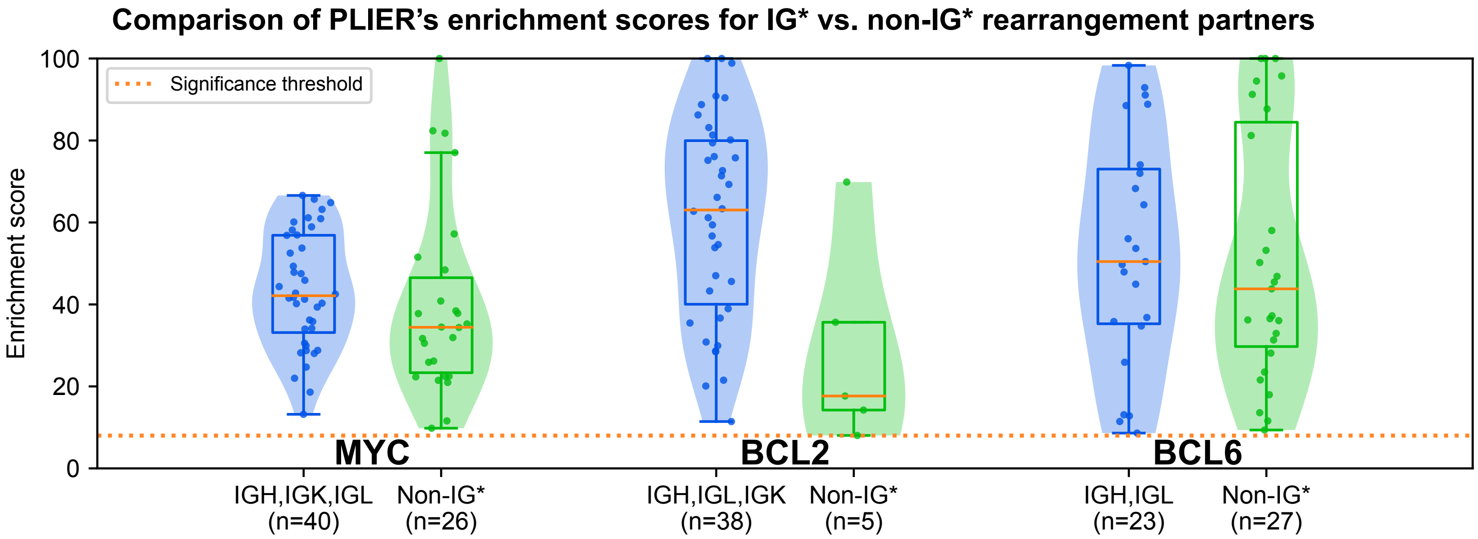

Suppl. Figure 9. Enrichment score of IG* rearranged partners vs. non-IG* rearranged partners. IG* rearranged partners are generally showing higher enrichment scores. Each boxplot (or the violin plot underneath) refers to the distribution of enrichment scores identified for a particular category of partners: IG* partners are shown in blue and non-IG* partners are shown in green. Each box extends from the lower (Q1) to upper quartile (Q3) values of the depicted data, with a line drawn at the median. Number of partners in each category is shown underneath each label (n=X) and also shown by dots "." that are overlayed on the boxplot. In addition, the lower whisker in each box plot depicts the Q1 - 1.5 * IQR position of the corresponding data distribution, where IQR is defined as the interquartile range (Q3-Q1). Similarly, the upper whisker depicts the Q3 + 1.5 * IQR position of the corresponding data distribution. The significance threshold (enrichment score > 8.0) is shown by a red dashed line.

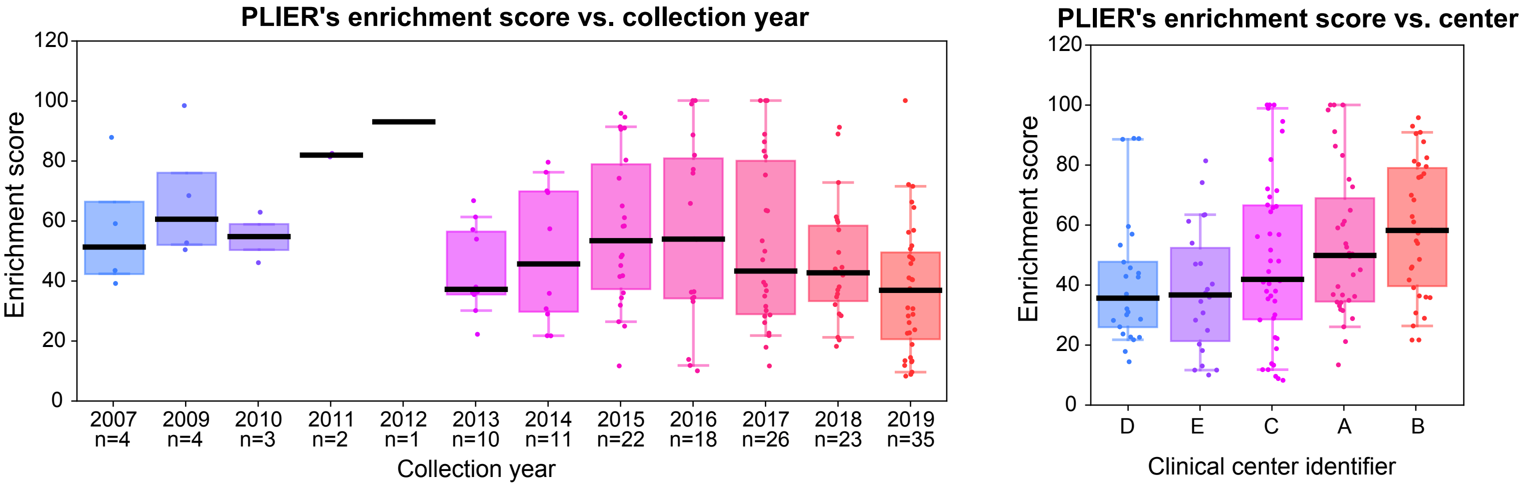

Suppl. Figure 10. PLIER's enrichment scores compared to clinical variables.
In this plot, the left panel indicates the distribution of enrichment scores of samples collected in a certain year (x-axis). The number of samples collected in each year is mentioned underneath each x-tick (i.e., n=X). The right panel indicates the distribution of enrichment score across clinical centers that contributed samples to our study. X-ticks refers to the center's identifier (see Suppl. Table 2 for center identifiers). Each boxe of a given boxplot extends from the lower (Q1) to upper quartile (Q3) values of the depicted data, with a line drawn at the median in black. In addition, the lower whisker in each box plot depicts the Q1 - 1.5 * IQR position of the corresponding data distribution, where IQR is defined as the interquartile range (Q3-Q1). Similarly, the upper whisker depicts the Q3 + 1.5 * IQR position of the corresponding data distribution.

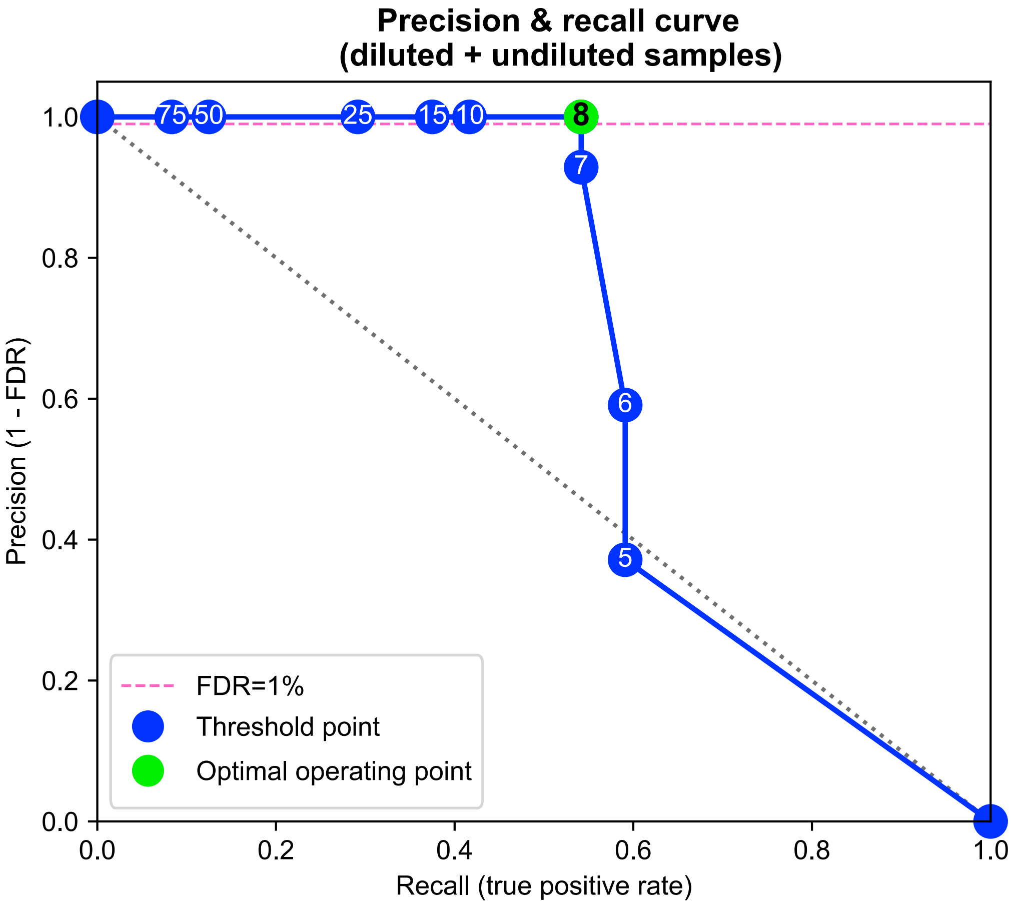

Suppl. Figure 11. Receiver operative curve of PLIER calls across different significance threshold.
With FDR below 1%, the threshold of 8.0 is chosen as an optimal point to determine significance. In this plot, the x-axis indicates the recall and the y-axis refers to the precision of PLIER calls made for a certain significance threshold (the threshold is shown within the circles in the plot). Thresholds with minor change of precision and recall are not shown. For this analysis, we included all dilution samples with their undiluted counterparts. The sample/target genes for which no FISH data was available were excluded from this analysis.

Suppl. Table 1. 4C Primers used to produce 4C experiments in this study.

Name	Sequence	Chr	Loc	Gene
BCL2_60920438_Fw	TACACGACGCTCTTCCGATCTGTAAGTGGCAGCTACACCAT	18	60920438	
BCL2_60920438_Rv	ACTGGAGTTCAGACGTGTGCTCTTCCGATCTAGTCCATACTGCTCACACCT			
BCL2_60880740_Fw	TACACGACGCTCTTCCGATCTAGGAGTTAAAACGAGAACCC	18	60880740	
BCL2_60880740_Rv	ACTGGAGTTCAGACGTGTGCTCTTCCGATCTGTGTGAGGACCTACATTGGT			
BCL2_25kbDS_Fw	TACACGACGCTCTTCCGATCTCTTTGGATAACCTGTTGCAT	18	60779113	
BCL2_25kbDS_Rv	ACTGGAGTTCAGACGTGTGCTCTTCCGATCTAAGTCAGATGGAATCCAACC			
BCL2_50kbDS_Fw	TACACGACGCTCTTCCGATCTCGTCCATCAGCTGTCTAAG	18	60746416	
BCL2_50kbDS_Rv	ACTGGAGTTCAGACGTGTGCTCTTCCGATCTTACATCCAGTTTGGCATACA			
BCL6_10kbDS_Fw	TACACGACGCTCTTCCGATCTGGACCAGTAGGAACCCTTAA	3	187432447	
BCL6_10kbDS_Rv	ACTGGAGTTCAGACGTGTGCTCTTCCGATCTAGTTGGTCTCCTATGGCTTT			
BCL6_150kbUS_Fw	TACACGACGCTCTTCCGATCTCTGTCAATTTAGGGAACAGC	3	187614849	
BCL6_150kbUS_Rv	ACTGGAGTTCAGACGTGTGCTCTTCCGATCTCCAGCGACTCTGATTAAAAG			
BCL6_15kbUS_Fw	TACACGACGCTCTTCCGATCTAGCAGAGTCCGGACATTT	3	187474752	
BCL6_15kbUS_Rv	ACTGGAGTTCAGACGTGTGCTCTTCCGATCTTATTGGAATGACGGATAACC			
BCL6_Fw	TACACGACGCTCTTCCGATCTAACTCAAACCCCAAGCAATG	3	187450285	
BCL6_Rv	ACTGGAGTTCAGACGTGTGCTCTTCCGATCTTAGTTACTGCAAAGGGGCTA			
BCL6_290kbUS_Fw	TACACGACGCTCTTCCGATCTCATTGCGGATTTGGATAC	3	187759922	
BCL6_290kbUS_Rv	ACTGGAGTTCAGACGTGTGCTCTTCCGATCTTAAAATTTGAAGGCCTTTCA			
MYC_128830560_Fw	TACACGACGCTCTTCCGATCTGGTTCATCAGCAGGATTTAT	8	128830560	
MYC_128830560_Rv	ACTGGAGTTCAGACGTGTGCTCTTCCGATCTAAGTCTAAAATGGGAAGCCT			
MYC_128773166_Fw	TACACGACGCTCTTCCGATCTTTGTTCATAAAACTGCGAGA	8	128773166	
MYC_128773166_Rv	ACTGGAGTTCAGACGTGTGCTCTTCCGATCTCCCCAAATCTACTACGTCAG			
MYC_100kbDS_Fw	TACACGACGCTCTTCCGATCTCTAGGCTGCAGTCCTGAAT	8	128851246	
MYC_100kbDS_Rv	ACTGGAGTTCAGACGTGTGCTCTTCCGATCTCACCCATATAATAAAAGGCG			
MYC_200kbUS_Fw	TACACGACGCTCTTCCGATCTATACAATGCCTCAGATGCTT	8	128562344	
MYC_200kbUS_Rv	ACTGGAGTTCAGACGTGTGCTCTTCCGATCTAAAGCCAACATTCTAGCTTC			
MYC_20kbUS_Fw	TACACGACGCTCTTCCGATCTCACCTCAATCCTTGTTCAAG	8	128735995	
MYC_20kbUS_Rv	ACTGGAGTTCAGACGTGTGCTCTTCCGATCTAAGAATGGTGCTTTGGATTA			
IgH_106441535_Fw	TACACGACGCTCTTCCGATCTCAGTCCAAATGTTAGCATCA	14	106441342	
IgH_106441535_Rv	ACTGGAGTTCAGACGTGTGCTCTTCCGATCTGTAGGTTCTGAGCATCCCTT			
IgH_Eu_Fw	TACACGACGCTCTTCCGATCTACACGTATACAGGCAGCAC	14	106320062	
IgH_Eu_Rv	ACTGGAGTTCAGACGTGTGCTCTTCCGATCTGTGCACTCAGGACCAGTATC			
IgH_RR1_Fw	TACACGACGCTCTTCCGATCTGAGGTCTCCCCATTTGCTC	14	106166687	
IgH_RR1_Rv	ACTGGAGTTCAGACGTGTGCTCTTCCGATCTACTCTGAGCCTCCAGGAAAC			
IgK_3E_Fw2	TACACGACGCTCTTCCGATCTGTAAAGAACTGAGGATTGGC	2	89130252	
IgK_3E_Rv2	ACTGGAGTTCAGACGTGTGCTCTTCCGATCTAACACCTTCGTGCATAAATT			
IgK_89131583_Fw	TACACGACGCTCTTCCGATCTAGGCTCTTACCAGGACTCAT	2	89131488	
IgK_89131583_Rv	ACTGGAGTTCAGACGTGTGCTCTTCCGATCTGAGCTAGCTGACAGGATGAC			
IgL_23284838_Fw	TACACGACGCTCTTCCGATCTTTAGTGTTGAGCAAGCTTCA	22	23284711	
IgL_23284838_Rv	ACTGGAGTTCAGACGTGTGCTCTTCCGATCTGAGGCTATAGGAAGATTGCC			
IgL_23403654_Fw	TACACGACGCTCTTCCGATCTTCAAGGGTTGATTAGTTTGC	22	23403826	
IgL_23403654_Rv	ACTGGAGTTCAGACGTGTGCTCTTCCGATCTGTACAGAAAGCTCATCACGG			
chr3_176795170_Fw	TACACGACGCTCTTCCGATCTTCTTCAAGTGAGTATGCTGGT			TBL1XR1
chr3_176795170_Rv	ACTGGAGTTCAGACGTGTGCTCTTCCGATCTCTTTTCCTCAAGGACTCCAT			TBL1XR1
chr3_176800784_Fw	TACACGACGCTCTTCCGATCTTCAAAAACCTGTTCGAAATT			TBL1XR1
chr3_176800784_Rv	ACTGGAGTTCAGACGTGTGCTCTTCCGATCTTTAGCGAGATTGGATTGATT			TBL1XR1
chr3_176699284_Fw	TACACGACGCTCTTCCGATCTAAATACAGCCTATGCCTTCC			TBL1XR1
chr3_176699284_Rv	ACTGGAGTTCAGACGTGTGCTCTTCCGATCTTGATGAGGAAGTGGGTAATC			TBL1XR1
chr4_86570997_Fw	TACACGACGCTCTTCCGATCTTTTCCAATATACGGTGTAAATTT			ARHGAP24
chr4_86570997_Rv	ACTGGAGTTCAGACGTGTGCTCTTCCGATCTGGCATTATGAAGCCTAATGA			ARHGAP24

Suppl. Table 2. Comparison FFPE-4C and FFPE-TLC results. Rearrangements shown in bold indicate that an additional 4C experiment is performed and confirmed the rearrangment reciprocally.

Sample	type	MYC_4C	BCL2_4C	BCL6_4C	MYC_TLC	BCL2_TLC	BCL6_TLC
F044 (A)	case	IGH-MYC			MYC-IGH		
F050 (A)	case	IGH-MYC			MYC-IGH<br>MYC-chr12:70mb		
F053 (A)	case	IGH-MYC			MYC-IGH		
F055 (A)	case	IGH-MYC			MYC-IGH		
F059 (A)	case	IGH-MYC			MYC-IGH		
F067 (A)	case	IGH-MYC			MYC-IGH		BCL6-IGL, BCL6-KYNU
F016 (A)	case	MYC-chr8:138mb	IGH-BCL2		MYC-chr8:43mb	BCL2-IGH	
F057 (A)	case		IGH-BCL2			BCL2-IGH	
F020 (A)	case		IGH-BCL2			BCL2-IGH	
F046 (A)	case		IGH-BCL2			BCL2-IGH	
F039 (A)	case		IGH-BCL2	BCL6-BACH2		BCL2-IGH	BCL6-BACH2
F014 (A)	case			IGH-BCL6			BCL6-IGH
F069 (A)	case			**BCL6-ARHGAP24**			BCL6-ARHGAP24
F037 (A)	case			BCL6-IGL			BCL6-IGL
F049 (A)	case			**BCL6-TBL1XR1**			BCL6-TBL1XR1
F025 (A)	case			IGH-BCL6			BCL6-IGH
F038 (A)	case			IGH-BCL6			BCL6-IGH
F040 (A)	case			IGH-BCL6			BCL6-IGH
F045 (A)	case			IGL-BCL6			BCL6-IGL
F036 (A)	case						
F054 (A)	case					BCL2-IGH	
F001 (A)	case						
F015 (A)	case						
F021 (A)	case						
F023 (A)	case						
F024 (A)	case						
F041 (A)	case						
F042 (A)	case						
F043 (A)	case						
F052 (A)	case						
F056 (A)	case						
F058 (A)	case						
F005 (A)	control						
F006 (A)	control						
F007 (A)	control						
F009 (A)	control						
F010 (A)	control						
F011 (A)	control						
F012 (A)	control						
F013 (A)	control						
F017 (A)	control						
F018 (A)	control						
F019 (A)	control						
F022 (A)	control						
F026 (A)	control						
F027 (A)	control						
F029 (A)	control						

Suppl. Table 3. Result of dilutions coverage vs. enrichment score experiment.

Sample	**Gene**	**Dilution**	**#False positives**		**PLIER**	**z-score (SV)**
			**Coverage**	**Enrichement**		
F46	BCL2	0.2%	161	0	x	2.5
F73	BCL2	0.2%	260	0	x	1.0
F37	BCL6	0.2%	339	0	x	2.2
F45	BCL6	0.2%	87	0	x	2.1
F50	MYC	0.2%	196	0	x	1.4
F59	MYC	0.2%	83	0	x	3.1
F46	BCL2	1%	3	0	found	9.0
F73	BCL2	1%	343	0	x	1.4
F37	BCL6	1%	19	0	x	3.5
F45	BCL6	1%	14	0	x	3.4
F50	MYC	1%	38	0	x	2.1
F59	MYC	1%	17	0	x	3.9
F46	BCL2	5%	0	0	found	44.1
F73	BCL2	5%	13	0	found	8.8
F37	BCL6	5%	1	0	found	15.1
F45	BCL6	5%	0	0	found	16.7
F50	MYC	5%	8	0	found	8.2
F59	MYC	5%	2	0	found	15.5
F46	BCL2	undiluted	0	0	found	101.8
F73	BCL2	undiluted	0	0	found	82.7
F37	BCL6	undiluted	0	0	found	52.1
F45	BCL6	undiluted	0	0	found	60.9
F50	MYC	undiluted	0	0	found	41.6
F59	MYC	undiluted	0	0	found	96.4

Suppl. Table 4. List of patents that are related to this work.

Patent applicant	Inventors	Application #	Status	Aspect of manuscript covered
Hubrecht Institute-KNAW/Cergentis	Max van Min, Wouter de Laat	EP2591125A2	Granted	TLA, target enrichment of proximity ligated molecules
Hubrecht Institute-KNAW	Amin Allahyar, Wouter de Laat, Erik Splinter	P6091185EP	Application	PLIER peak calling
Cergentis	Joost Swennenhuis, Erik Splinter	P2010494.9	Application	FFPE-TLC reverse crosslinking step
Cergentis	Mehmet Yilmaz, Erik Splinter	P2010492.3	Application	FFPE-TLC tissue permeabilization step
ErasmusMC	Frank Grosveld, Wouter de Laat	WO/2008/084405	Granted	4C, Target enrichment of proximity ligated DNA molecules
ErasmusMC	Frank Grosveld, Wouter de Laat	WO/2007/004057	Granted	4C experiments

Suppl. Table 5. Samples analyzed with Capture-NGS

Sample number	Covaris	Covaris Input (ng)	Capture panel	HISeq	insertsize (bp)
F72_Sample 1	S2	690	V2	2500	125
F73_Sample 2	S2	497	V2	2500	125
F74_Sample 3	S2	321	V1	2500	125
F75_Sample 4	ME220	250	V2	4000	150
F76_Sample 5	S2	510	V1	2500	125
F77_Sample 6	S2	Unknown (15ul DNA isolate)	V2	2500	125
F78_Sample 7	S2	282	V1	2500	125
F188_Sample 8	S2	419	V2	2500	125
F189_Sample 9	S2	Unknown (15ul DNA isolate)	V2	2500	125
F190_Sample 10	ME220	250	V2 & V3	2500 & 4000	150
F191_Sample 11	S2	250	V2	2500	125
F192_Sample 12	S2	570	V2	2500	125
F193_Sample 13	S2	336	V2	2500	125
F194_Sample 14	S2	Unknown (15ul DNA isolate)	V2	2500	125
F195_Sample 15	S2	600	V1	2500	125
F196_Sample 16	S2	250	V1	2500	125
F197_Sample 17	S2	Unknown (15ul DNA isolate)	V2	2500	125
F198_Sample 18	S2	Unknown (15ul DNA isolate)	V2	2500	125
F199_Sample 19	S2	Unknown (15ul DNA isolate)	V2	2500	125

Suppl. Table 6. Capture-NGS probe details

V1	Order ID	187705000		
Chrom	Start	Stop	Gene	Area size
2	89157133	89184926	IGK	27793
3	176738541	176782810	TBL1XR1	44269
3	187439164	187463513	BCL6	24349
3	189349294	189612301	TP63	263007
7	13935480	14028687	ETV1	93207
8	128746035	128761810	MYC	15775
9	5450499	5510803	CD274	60304
9	5510804	5571282	PDCD1LG2	60478
9	36838530	37034476	PAX5	195946
11	69455872	69469242	CCND1	13370
12	11802787	12048325	ETV6	245538
14	106032614	107288051	IGH	1255437
16	10878740	11017182	CIITA	138442
18	60760000	60987282	BCL2	227282
22	23,229,960	23,238,013	IGLL5	8053
22	23238014	23264766	IGL	26752
22	23522551	23660224	BCR	137673
22	23,915,500	23,922,400	IGLL1	6900
total size				2,844,575

V2	Order ID	43712		
Chrom	Start	Stop	Gene	Area size
2	89156500	89162000	IGK	5500
3	176738000	176783000	TBL1XR1	45000
3	187439000	187475000	BCL6	36000
3	187705000	187750000	BCL6	45000
3	189455000	189527000	TP63	72000
8	128446000	128905000	MYC	459000
8	129103000	129404000	MYC	301000
9	5450000	5471000	CD274	21000
9	5503000	5572000	PDCD1LG2	69000
9	37020000	37036000	PAX5	16000
14	106050000	106400000	IGH	350000
16	10970000	10975000	CIITA	5000
16	10992500	10996000	CIITA	3500
18	60760000	60797000	BCL2	37000
18	60984000	60996600	BCL2	12600
22	23235000	23265000	IGL	30000
total size				1,507,600

V3	Order ID	1000002633		
Chrom	Start	Stop	Gene	Area size
2	89156500	89165500	IGK	9000
3	176738000	176915000	TBL1XR1	177000
3	187455000	187475000	BCL6	20000
3	187660000	187750000	BCL6	90000
3	189455000	189527000	TP63	72000
8	128510000	129220000	MYC	710000
9	5450000	5471000	CD274	21000
9	5471000	5503000	CD274-PDCD1LG2	32000
9	5503000	5572000	PDCD1LG2	69000
9	37020000	37036000	PAX5	16000
14	106053000	106058000	IGH	5000
14	106064000	106074000	IGH	10000
14	106089000	106100000	IGH	11000
14	106108000	106118000	IGH	10000
14	106173000	106180000	IGH	7000
14	106200000	106220000	IGH	20000
14	106232000	106243000	IGH	11000
14	106298000	106400000	IGH	102000
16	10970000	10975000	CIITA	5000
16	10992500	10996000	CIITA	3500
18	60760000	60797000	BCL2	37000
18	60984000	60996600	BCL2	12600
22	23235000	23265000	IGL	30000
total size				1,480,100
