# Supplementary material for: Robust detection of translocations in lymphoma FFPE samples using targeted locus capture-based sequencing
Source: Nat Commun. 2021 Jun 7;12:3361. doi: 10.1038/s41467-021-23695-8 (PMC8184748; doi:10.1038/s41467-021-23695-8)
Supplement: Supplementary file 3 — Reporting Summary [file 41467_2021_23695_MOESM3_ESM.pdf]

nature research

Corresponding author(s): Wouter de Laat and Erik Splinter

Last updated by author(s): Apr 29, 2021

Reporting Summary

Nature Research wishes to improve the reproducibility of the work that we publish. This form provides structure for consistency and transparency in reporting. For further information on Nature Research policies, see our Editorial Policies and the Editorial Policy Checklist.

Please do not complete any field with "not applicable" or n/a. Refer to the help text for what text to use if an item is not relevant to your study.
For final submission: please carefully check your responses for accuracy; you will not be able to make changes later.

Statistics

For all statistical analyses, confirm that the following items are present in the figure legend, table legend, main text, or Methods section.

n/a	Confirmed	
☐	☒	The exact sample size (*n*) for each experimental group/condition, given as a discrete number and unit of measurement
☐	☒	A statement on whether measurements were taken from distinct samples or whether the same sample was measured repeatedly
☒	☐	The statistical test(s) used AND whether they are one- or two-sided<br>*Only common tests should be described solely by name; describe more complex techniques in the Methods section.*
☐	☒	A description of all covariates tested
☐	☒	A description of any assumptions or corrections, such as tests of normality and adjustment for multiple comparisons
☐	☒	A full description of the statistical parameters including central tendency (e.g. means) or other basic estimates (e.g. regression coefficient) AND variation (e.g. standard deviation) or associated estimates of uncertainty (e.g. confidence intervals)
☒	☐	For null hypothesis testing, the test statistic (e.g. *F*, *t*, *r*) with confidence intervals, effect sizes, degrees of freedom and *P* value noted<br>*Give P values as exact values whenever suitable.*
☒	☐	For Bayesian analysis, information on the choice of priors and Markov chain Monte Carlo settings
☐	☒	For hierarchical and complex designs, identification of the appropriate level for tests and full reporting of outcomes
☒	☐	Estimates of effect sizes (e.g. Cohen's *d*, Pearson's *r*), indicating how they were calculated

Our web collection on statistics for biologists contains articles on many of the points above.

Software and code

Policy information about availability of computer code

Data collection	Paired-end sequencing was done on an Illumina Novaseq 6000 sequencing machine.
Data analysis	Sequenced reads from individual samples (i.e. patients) were mapped to the human genome (hg19) using BWA-MEM (version: 0.7.17-r1188; settings: -SP -k12 -A2 -B3) in paired-end mode. The demultiplexing and other related preprocessing steps are done using in-house scripts (see: https://github.com/deLaatLab/PLIER). The rearrangements were identified using PLIER: https://github.com/deLaatLab/PLIER.<br><br>HiC libraries (F50dil5, F59dil5, F209, F197, F199, F67) were sequenced in an Illumina NovaSeq 6000 machine in 2x150bp paired-end mode. The corresponding FASTQ files were processed following the 4DN HiC processing pipeline recommendations 41. The resulting pairix files were accessed by Pairix 42 (v0.3.7) to produce the butterfly plots (shown in Suppl. Figure 8) by visualizing the captured interactions between two regions in the genome (target vs. rearranged partner) in a heatmap similar to the standard HiC matrices. The bin width for each butterfly plot is chosen as 20kb (or 50kb if the plot was too sparse).<br><br>Fusion-read mappability: The identified breakpoint coordinates from the fusion reads were used in the mappability analysis to extract the corresponding sequences from the reference genome. In total 347 sequences of 151 bp (equal to the sequencing read length) upstream and downstream of the breakpoints were extracted from the reference genome. These 347 sequences were aligned using BLASTn (version: 2.8.1; settings: -perc_identity 80 -dust no -evalue 0.1) at different sequence lengths from 20 to 151, using a step size of 1 bp. The blast results were parsed to count the sequences with exact hits at each length; if exactly one hit, the sequence is considered unique, if multiple hits the sequence is considered non-unique. The fraction of non-unique sequences was plotted in a bar graph.

For manuscripts utilizing custom algorithms or software that are central to the research but not yet described in published literature, software must be made available to editors and reviewers. We strongly encourage code deposition in a community repository (e.g. GitHub). See the Nature Research guidelines for submitting code & software for further information.

Data

Policy information about availability of data

All manuscripts must include a data availability statement. This statement should provide the following information, where applicable:

- Accession codes, unique identifiers, or web links for publicly available datasets
- A list of figures that have associated raw data
- A description of any restrictions on data availability

All sequencing data used in this study were mapped to the reference genome (hg19) and are available through the European Genome-phenome Archive (EGA study ID: EGAS00001004760). Of note, to protect patients' privacy, this submission is fully anonymized and is protected by the UMC Data Access Committee. A formal approval is needed to download the data.
Figure 1-5 as well as all supplementary figures are produced using the data above.

Field-specific reporting

Please select the one below that is the best fit for your research. If you are not sure, read the appropriate sections before making your selection.

☒ Life sciences ☐ Behavioural & social sciences ☐ Ecological, evolutionary & environmental sciences

Life sciences study design

All studies must disclose on these points even when the disclosure is negative.

Sample size	The sample size is not determined by any statistical tests. Our preliminary analyses with 20 samples (a mixture of FFPE-4C and FFPE-TLC libraries, including diluted samples) showed that the hyperparameters of PLIER can be trained successfully using this set of samples, and further assessments by hold-out samples confirmed that this training is sufficient to identify the rearrangements even at 5% dilution. In total, we performed our study with 149 sequenced samples which was limited by the number of samples that each collaborating clinical center could contribute.
Data exclusions	No data is excluded from this study.
Replication	For several samples, we included (biological and technical) replicates. In total, we performed 13 biological replicates, and 2 technical replicates (see Supplementary Table 2 for details). All attempts for calling rearrangements in the replicates were successful, with exception of an IGH viewpoint designed on F212 sample, which failed to identify the MYC gene in the replicate (F212b). Note that for this sample, the MYC-IGH rearrangement was identified in both samples (F212 and F212b) when looking from MYC viewpoint.
Randomization	Randomization is performed in PLIER to estimate the enrichment scores. No other randomization is performed in the study as such a procedure was not applicable in those parts.
Blinding	This study is performed in a blind fashion were sample diagnosis were hidden from the authors.

Reporting for specific materials, systems and methods

We require information from authors about some types of materials, experimental systems and methods used in many studies. Here, indicate whether each material, system or method listed is relevant to your study. If you are not sure if a list item applies to your research, read the appropriate section before selecting a response.

Materials & experimental systems

n/a	Involved in the study	
☒	☐	Antibodies
☒	☐	Eukaryotic cell lines
☒	☐	Palaeontology and archaeology
☒	☐	Animals and other organisms
☐	☒	Human research participants
☒	☐	Clinical data
☒	☐	Dual use research of concern

Methods

n/a	Involved in the study	
☒	☐	ChIP-seq
☒	☐	Flow cytometry
☒	☐	MRI-based neuroimaging

Human research participants

Policy information about studies involving human research participants

Population characteristics	Apart from negative control samples, other samples used in this study were chosen specifically to contain at least one

Population characteristics	rearrangement in MYC, BCL2 or BCL6 gene. The contributing clinical centers shared no further information regarding the ethnicity of the utilized samples with the authors of this work.
Recruitment	Considering the rarity of the rearrangement events occurring in MYC, BCL2 and BCL6 genes in lymphoma tumors, samples were specifically selected to contain these rearrangements. In addition, 20 negative control samples were included (in a "blind" manner) that were expected to be devoid of rearrangement in MYC, BCL2 and BCL6. The total number of negative control and their sample IDs were kept hidden from the contributing authors until the final stage of the study.
Ethics oversight	The use of tissue specimens and associated data in this study was approved by the Medical Ethical Committee of the University Medical Center Groningen (RR 201800551) for explorative research, Medical Ethical Committee of LabPON under “nader gebruik geen bezwaar”, the TcBio of UMCU as “gebruik van restmateriaal”, TcBio of VUMC/AUMC under “nader gebruik geen bezwaar” and the Medical Ethical Committee of LUMC under code of conduct of secondary use of tissues.

Note that full information on the approval of the study protocol must also be provided in the manuscript.

This checklist template is licensed under a Creative Commons Attribution 4.0 International License, which permits use, sharing, adaptation, distribution and reproduction in any medium or format, as long as you give appropriate credit to the original author(s) and the source, provide a link to the Creative Commons license, and indicate if changes were made. The images or other third party material in this article are included in the article's Creative Commons license, unless indicated otherwise in a credit line to the material. If material is not included in the article's Creative Commons license and your intended use is not permitted by statutory regulation or exceeds the permitted use, you will need to obtain permission directly from the copyright holder. To view a copy of this license, visit http://creativecommons.org/licenses/by/4.0/
